# Smoking and prevalence of COVID-19: Evidence from studies from January 2020 – May 2020

**DOI:** 10.3934/publichealth.2023038

**Published:** 2023-06-19

**Authors:** Rafia Butt, Rehan Ahmad Khan Sherwani, Muhammad Aslam, Mohammed Albassam

**Affiliations:** 1 College of Statistical Sciences, University of the Punjab Lahore, Pakistan; 2 Department of Statistics, Faculty of Science, King Abdulaziz University, Jeddah 21551, Saudi Arabia

**Keywords:** COVID-19, PRISMA, pooled proportion, forest plot, smoking, prevalence, effect size

## Abstract

It is well-known that smoking tobacco harms the respiratory system and can lead to various health problems. Regarding COVID-19, a respiratory illness caused by the novel coronavirus SARS-CoV-2, smoking may have implications for both the risk of infection and the severity of the disease. Several studies have explored the association between smoking and COVID-19. However, findings have been somewhat inconsistent and vary from region to region for sample size. This article aims to study the prevalence of COVID-19 among those affected with their ongoing smoking history by computing pooled estimates of the published research. Fixed effect meta-analysis by following the guidelines of PRISMA has been carried out on 34 studies. The patients with confirmed RT-PCR and CT-scan were included, a total of 13,368; The studies' quality assessment was performed according to the Appraisal Checklist recommended by the Joanna Briggs Institute. The effect sizes of the published research are presented in the form of pooled estimates with their respective confidence intervals. Forest plots are used to represent the effect size graphically. Current smokers' effect sizes are 0.12 (CI = 0.11–0.12); for non-smokers, it is estimated to be 0.88 (CI = 0.88–0.89). The heterogeneity statistic *I*^2^ describes 0% of the total variation, meaning no heterogeneity among studies exists. A higher prevalence of COVID-19 among non-smokers is observed than the smokers.

## 1. Introduction

COVID-19 is the newly found virus belonging to the family of coronaviruses. It is a virus typically affecting the respiratory tract of humans and other mammals that initially broke in Wuhan Wholesale Seafood Market, China, in late December 2019 [1]–[3]. It has been declared a world pandemic by the World Health Organization (WHO) [4]. The novel Coronavirus is COVID-19, Coronavirus Disease of 2019. It is a contagious virus that spreads through the saliva and the fluid discharge through the mouth and nose while coughing and sneezing. It initially spread through the Wuhan Seafood Market, China, where wild animals were sold [5].

COVID-19 affects the upper respiratory tract first, which causes pneumonia and Acute Respiratory Distress Syndrome (ARDS). Adults are more susceptible to COVID-19. It can cause illnesses ranging from mild to severe. This is why the mortality rate due to COVID-19 is higher in elders with at least one underlying disease [6]. In the Centers for Disease Control and Prevention Institute report, patients diagnosed with COVID-19 have 9.2% of respiratory disease [7]. The most common comorbidities found in the confirmed COVID-19 patients were Asthma and Chronic obstructive pulmonary disease (COPD) in adults aged ≥18 years [8]. It has been observed that COVID-19 progresses more severely in COPD patients [9].

Smoking is a leading cause of lung cancer and adversely affects lung health [10],[11]. Use of Tobacco plays a vital role in the cause and development of COPD, and it may also have a common effect on symptoms [12]. Tobacco smoking causes 90% of lung cancer cases and is considered a significant inevitable risk of global death [13]. Smoking is associated with an increased risk of disease severity and death for COVID-19 patients [14].

COVID-19 majorly spreads through released respiratory droplets when the infected patients cough, sneeze and talk. When these droplets land on any surface, or the person nearby breathes, they transmit from the infected person to the nearby uninfected person, which is how it spreads from person to person. It has become a global concern about the impact of smoking on disease severity and susceptibility. Smoking has been considered an established risk factor for respiratory infections [15]. The immune system could weaken by smoking and damage the lungs, making it harder for the body to resist the infection of COVID-19. Smoking also increases the likelihood of virus transmission by using fingers to mouth for smoking, increasing the risk of infection transmission from contaminated surfaces. Thus, it is crucial to study whether smoking plays a vital role in the prevalence of COVID-19. If yes, government policies must address this problem to avoid adverse outcomes.

For several reasons, it is necessary to study the smoking history of a COVID-19 patient:

1. **Higher risk of severe illness:** It is believed that smoking causes many health issues, especially related to respiratory illness. So, smokers have a higher risk of severe illness and mortality due to COVID-19 because smoking damages the lungs and weaken the immune system. It makes it hard for the body to resist infections.

2. **To understand the disease progression:** It can help the health care providers better understand how the infection of COVID-19 progresses in smokers vs non-smokers. This can help in better treatment protocols and improved outcomes.

3. **Risk assessment:** By studying a patient's smoking history, healthcare providers assess the risk of severe infection and tailor the treatment accordingly.

So, it is important to study the smoking patterns of a COVID-19 patient. It gives important information for better understanding the disease progression, access the risk of disease and better provision of treatment.

Farsalinos et al. systematically reviewed whether electronic cigarettes have lower risk than tobacco cigarettes and can be used as an alternative. The study showed that electronic cigarettes are less harmful and can be used as an alternative to tobacco cigarette [16]. It can be concluded that how worse outcomes can be obtained from tobacco smoking.

Lippi et al. conducted a meta-analysis with five published research articles on COVID-19 severity, and the results showed that smoking is not contributing significantly towards the severity of COVID-19 (Odds Ratio = 1.69), 95% Confidence Interval: (0.41–6.92)) [17]. The heterogeneity of the study was found to be low among the studies, i.e., (*I*^2^ = 38%). Another meta-analysis involving 11 studies provides different results opposing the abovementioned results and conclusion. In this meta-analysis, the severity of COVID-19 was found to increase with Smoking (Odds Ratio = 1.97), 95% Confidence Interval: (0.95–4.10) with heterogeneity among the studies (*I*^2^ = 44%) [9].

Coccia et al. concluded the importance of vaccination and that optimal vaccination coverage is required to reduce COVID-19. Mortalities due to COVID-19 depend on factors like population size, virus transmission rate and vaccine efficacy. The authors concluded that higher vaccination coverage is needed in countries or places with a high transmission rate [18].

In China, during the post-pandemic era of COVID-19 among pregnant women, there was a higher prevalence of anxious and depressive symptoms than before the pandemic started. The study mentioned multiple risk factors, such as financial challenges, loss of a job, low social support and bad life events. Studies show that healthcare providers should give attention and care towards the mental health of pregnant ladies and their partners, especially during the pandemic [19].

Even in the presence of vaccines, non-pharmaceutical interventions like wearing masks and social distancing are critical to controlling the spread of COVID-19 [20].

Lower oxygen saturation percentage and arterial oxygen pressure are associated with poor outcomes, including long ICU stays and high ICU mortality rates. The study's outcomes suggest that monitoring the oxygen saturation percentage and arterial oxygen pressure levels would help determine the condition of COVID-19 patients admitted to ICUs [21].

Understanding the cellular and molecular mechanism that is the underlying cause of the development of ARDS in COVID-19 infected patients is important. This will give insights into the development of well-efficient therapies for this disease [22].

Delgado et al. study the transmission routes of different pathogenic microorganisms in the environment, such as households, healthcare facilities and public places. Authors suggested that practicing different environment decontamination strategies will effectively control the spread of COVID-19 and other pathogens, ventilation and disinfection [23]. During a pandemic, establishing a hyperacute stroke service is effective, but that requires sensitive planning and implementing infection control measures [24]. Benati et al. discussed the importance of effective governance and emphasized ensuring that vaccines are equally distributed everywhere, especially in low-income countries [25].

People currently smoking cigarettes are at a high risk of being infected with COVID-19 and may develop severe infection of COVID-19 as compared to non-smokers [26]. The available literature is divided on the association between smoking history and the prevalence, causes and severity of COVID-19.

The divided opinion might be due to limited research on the topic. The present research explored the association between Smoking and COVID-19 on a larger scale and considered 34 studies. We presented a systematic review and meta-analysis to more concisely explore the severity of COVID-19 confirmed patients with their ongoing smoking history.

## 2. Materials and methods

### 2.1. Database search and study selection

The analysis is performed according to the guidelines of Preferred Reporting Items for Systematic Reviews and Meta-Analyses, abbreviated as PRISMA [27]. The study involves published research articles from different countries, i.e., China (26 articles), the United States (7 articles) and Brazil (1 article). PubMed, Web of Science, Google Scholar, Jstor, etc., are used for search engines, and 34 articles were shortlisted from January 24, 2020, to May 11, 2020. Different keywords used for the search are “Sars-cov-2”, “COVID-19”, “Corona Virus”, “Wuhan Coronavirus”, “Severe acute respiratory syndrome coronavirus”, “2019-nCov” and “smoking”. All included articles are in English, and there is no language restriction. Commentaries, reports, case studies, editorial letters and reviews are excluded.

Initially, articles were screened by their titles, date of publication, abstracts and only those COVID-19 articles were retrieved, including adult smokers and non-smokers (18 years and older). Duplicate studies were removed, and the full text of eligible articles was examined. The articles should be relevant and address the same problem.

#### 2.1.1. Inclusion criteria

Meta-analysis sets some boundaries for including articles followed in the present research [28]:

1. Studies included in the analysis were available in full text and addressed the same research topic.

2. Studies were not outdated and they reported the number of COVID-19-confirmed adult patients aged 18.

3. The patients included in the study were confirmed with real-time PCR for COVID-19 or the World Health Organization (WHO) criteria.

4. The studies included demographic data, exposure history, signs and symptoms and smoking history of COVID-19-confirmed patients.

#### 2.1.2. Exclusion criteria

Like inclusion criteria, meta-analysis has also set some boundaries for exclusion criteria [28]:

1. Studies were excluded with different variables of interest and didn't include patients' smoking history.

2. Those studies excluded those that didn't report the COVID-19 confirmed cases.

3. COVID-19 studies about infants or specifically about pregnant women were excluded.

4. The study did not include individual case reviews or reports, letters to editors and news reports.

### 2.2. Data analysis

**Figure 1.**
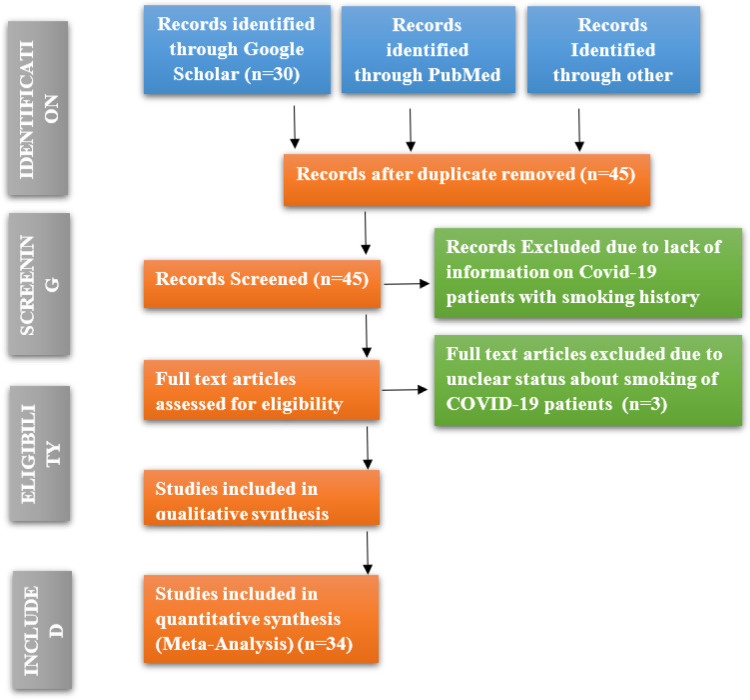
PRISMA flowchart.

The keywords described earlier in the initial search of International databases yielded 91 articles. Forty-five articles remained after excluding the duplicated articles, and 37 were assessed for eligibility. Eight articles were excluded due to a lack of information on COVID-19 patients with a smoking history, and three articles with full text were excluded due to the unclear status of smoking in COVID-19 patients. A total of 34 articles met the inclusion criteria discussed above. Guidelines by Preferred Reporting Items for Systematic Reviews and Meta-Analyses have been followed while conducting the analysis [29]. PRISMA diagram of the study selection procedure is mentioned in Figure 1.

Quality assessments of the studies are performed according to the Appraisal Checklist recommended by the Joanna Briggs Institute. The checklist comprised various questions for each study included in the analysis. The “yes” for each question obtained one point [30]. Meta-analysis results show the individual study's proportion of the outcome variable and a 95% Confidence Interval. In addition, an overall pooled estimate for the study variable is also presented, obtained using a fixed-effect model with a 95% Wald Confidence Interval. Statistics describing the total variation and weight for each study are also obtained. The forest plot is the graphical representation of the meta-analysis for proportions [31]. For count data, the metaprop command is used in Stata (14.1 version) to compute the combined or pooled proportions [32]. A fixed effect model has been used because all the studies included in the analysis have the same effect size, and there is no heterogeneity. The combined effect for each study is statistically significant (p < 0.05).

## 3. Results

**Figure 2.**
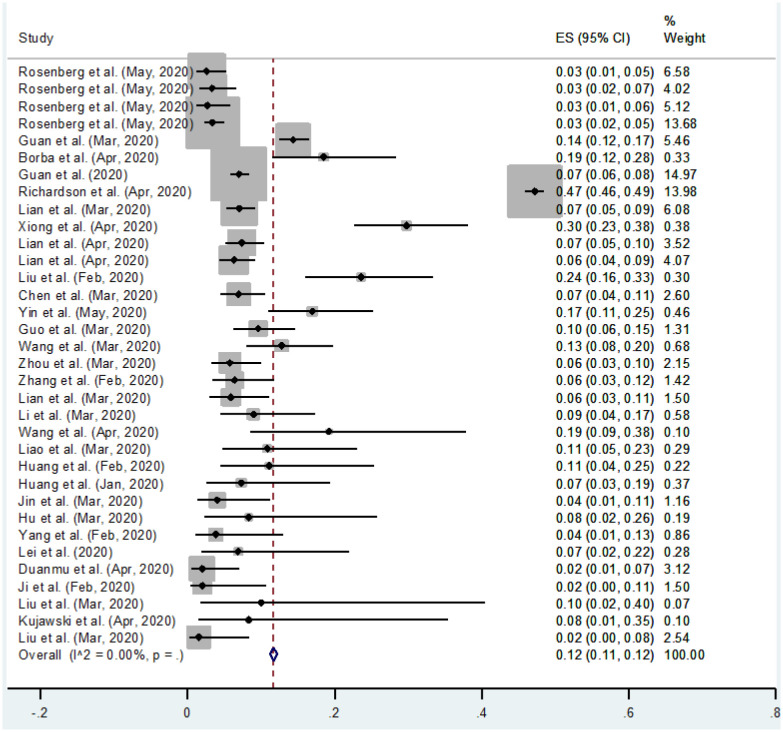
Forest plot of COVID-19 infected patients who currently smoke (ES = Effect size), (CI = Confidence Interval).

**Figure 3.**
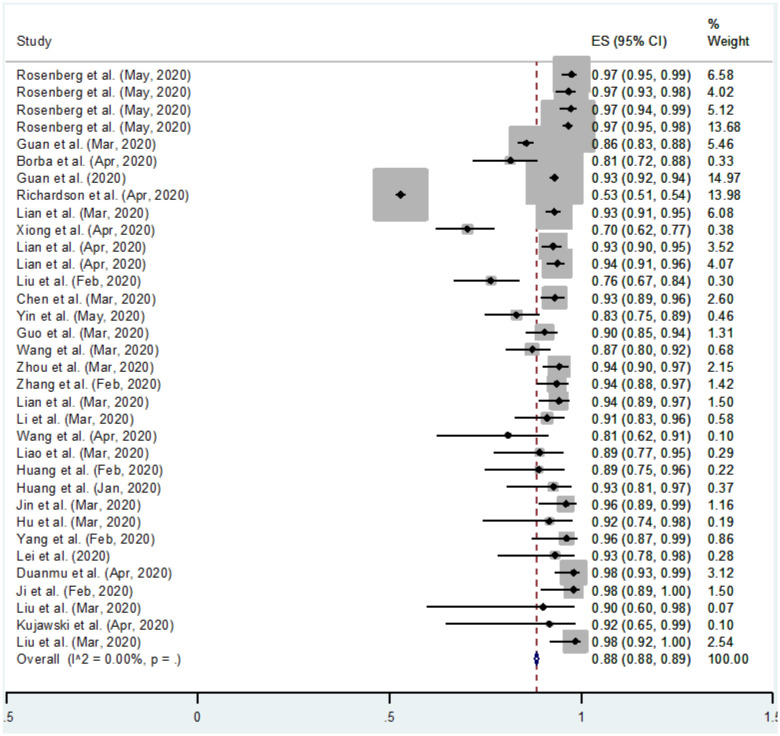
Forest plot of COVID-19 infected patients who do not smoke (ES= Effect size), (CI=Confidence Interval).

Meta-analysis has been carried out on 34 studies in which a total of 13,368 patients affected with COVID-19 confirmed with RT-PCR and CT-scan were included. Out of 13,368 patients, 3318 were smokers and 10,050 were non-smokers. The numerical results for pooled proportions for COVID-19 infected patients who currently smoke and do not smoke are shown in Tables 1 and 2, respectively. The results are presented graphically in Figures 2 and 3.

**Table 1.** Meta-analysis of the proportion of COVID-19-infected patients who currently smoke.

Study	ES	95% Conf. Interval	%	Weight
Rosenberg et al. (Mar, 2020)	0.03	0.01	0.05	6.58
Rosenberg et al. (Mar, 2020)	0.03	0.02	0.07	4.02
Rosenberg et al. (Mar, 2020)	0.03	0.01	0.06	5.12
Rosenberg et al. (Mar, 2020)	0.03	0.02	0.05	13.68
Guan et al. (Mar, 2020)	0.14	0.12	0.17	5.46
Borba et al. (Apr, 2020)	0.19	0.12	0.28	0.33
Guan et al. (2020)	0.07	0.06	0.08	14.97
Richardson et al. (2020)	0.47	0.46	0.49	13.98
Lian et al. (Mar, 2020)	0.07	0.05	0.09	6.08
Xiong et al. (Apr, 2020)	0.30	0.23	0.38	0.38
Lian et al. (Apr, 2020)	0.07	0.05	0.10	3.52
Lian et al. (Apr, 2020)	0.06	0.04	0.09	4.07
Liu et al. (Feb, 2020)	0.24	0.16	0.33	0.30
Chen et al. (Mar, 2020)	0.07	0.04	0.11	2.60
Yin et al. (May, 2020)	0.17	0.11	0.25	0.46
Guo et al. (Mar, 2020)	0.10	0.06	0.15	1.31
Wang et al. (Mar, 2020)	0.13	0.08	0.20	0.68
Zhou et al. (Mar, 2020)	0.06	0.03	0.10	2.15
Zhang et al. (Feb, 2020)	0.06	0.03	0.12	1.42
Lian et al. (Mar, 2020)	0.06	0.03	0.11	1.50
Li et al. (Mar, 2020)	0.09	0.04	0.17	0.58
Wang et al. (Apr, 2020)	0.19	0.09	0.38	0.10
Liao et al. (Mar, 2020)	0.11	0.05	0.23	0.29
Huang et al. (Feb, 2020)	0.11	0.04	0.25	0.22
Huang et al. (Jan, 2020)	0.07	0.03	0.19	0.37
Jin et al. (Mar, 2020)	0.04	0.01	0.11	1.16
Hu et al. (Mar, 2020)	0.08	0.02	0.26	0.19
Yang et al. (Feb, 2020)	0.04	0.01	0.13	0.86
Lei et al. (2020)	0.07	0.02	0.22	0.28
Duanmu et al. (Apr, 2020)	0.02	0.01	0.07	3.12
Ji et al. (Feb, 2020)	0.02	0.00	0.11	1.50
Liu et al. (Mar, 2020)	0.10	0.02	0.40	0.07
Kujawski et al. (Apr, 2020)	0.08	0.01	0.35	0.10
Liu et al. (Mar, 2020)	0.02	0.00	0.08	2.54
Fixed pooled ES	0.12	0.11	0.12	100.00

*Note: ES = 0; z = 47.42; p = 0.00.

Since smoking leaves a destructive impact on the lungs, people who usually do smoking have underlying lung disease. Breathing problem was one of the most faced complications by COVID-19-infected patients [33]–[35]. In this analysis, it is desired to check the prevalence of COVID-19 in smokers vs non-smokers.

The forest plot shown in Figure 2 is the graphical representation of the Meta-analysis for the proportion of COVID-19 patients who smoke. The results in Table 1 and Figure 2 show the individual study's proportion of the outcome variable and a 95% Confidence Interval. In addition, an overall pooled estimate for the study variable is also presented. The overall pooled estimate of a total sample size for 34 studies of a study variable “COVID-19 infected patients who smoke” is 0.12, obtained using a fixed effect model with a 95% Wald Confidence Interval. *I*^2^ statistic describes 0% of the total variation, meaning no heterogeneity exists.

**Table 2.** Meta-analysis of the proportion of COVID-19-infected patients who do not smoke.

Study	ES	95% Conf. Interval	%	Weight
Rosenberg et al. (Mar, 2020)	0.97	0.95	0.99	6.58
Rosenberg et al. (Mar, 2020)	0.97	0.93	0.98	4.02
Rosenberg et al. (Mar, 2020)	0.97	0.94	0.99	5.12
Rosenberg et al. (Mar, 2020)	0.97	0.95	0.98	13.68
Guan et al. (Mar, 2020)	0.86	0.83	0.88	5.46
Borba et al. (Apr, 2020)	0.81	0.72	0.88	0.33
Guan et al. (2020)	0.93	0.92	0.94	14.97
Richardson et al. (2020)	0.53	0.51	0.54	13.98
Lian et al. (Mar, 2020)	0.93	0.91	0.95	6.08
Xiong et al. (Apr, 2020)	0.70	0.62	0.77	0.38
Lian et al. (Apr, 2020)	0.93	0.90	0.95	3.52
Lian et al. (Apr, 2020)	0.94	0.91	0.96	4.07
Liu et al. (Feb, 2020)	0.76	0.67	0.84	0.30
Chen et al. (Mar, 2020)	0.93	0.89	0.96	2.60
Yin et al. (May, 2020)	0.83	0.75	0.89	0.46
Guo et al. (Mar, 2020)	0.90	0.85	0.94	1.31
Wang et al. (Mar, 2020)	0.87	0.80	0.92	0.68
Zhou et al. (Mar, 2020)	0.94	0.90	0.97	2.15
Zhang et al. (Feb, 2020)	0.94	0.88	0.97	1.42
Lian et al. (Mar, 2020)	0.94	0.89	0.97	1.50
Li et al. (Mar, 2020)	0.91	0.83	0.96	0.58
Wang et al. (Apr, 2020)	0.81	0.62	0.91	0.10
Liao et al. (Mar, 2020)	0.89	0.77	0.95	0.29
Huang et al. (Feb, 2020)	0.89	0.75	0.96	0.22
Huang et al. (Jan, 2020)	0.93	0.81	0.97	0.37
Jin et al. (Mar, 2020)	0.96	0.89	0.99	1.16
Hu et al. (Mar, 2020)	0.92	0.74	0.98	0.19
Yang et al. (Feb, 2020)	0.96	0.87	0.99	0.86
Lei et al. (2020)	0.93	0.78	0.98	0.28
Duanmu et al. (Apr, 2020)	0.98	0.93	0.99	3.12
Ji et al. (Feb, 2020)	0.98	0.89	1.00	1.50
Liu et al. (Mar, 2020)	0.90	0.60	0.98	0.07
Kujawski et al. (Apr, 2020)	0.92	0.65	0.99	0.10
Liu et al. (Mar, 2020)	0.98	0.92	1.00	2.54
Fixed pooled ES	0.88	0.88	0.89	100.00

*Note: ES = 0; z = 357.03; p = 0.00.

The last column in Table 1 and Figure 2 show the weight for each study. Moreover, the combined effect is also statistically significant (p < 0.05). The confidence intervals on the right side of zero show that the studies have a statistically significant positive effect. The confidence interval of the combined effect size does not include zero, showing that the combined effect is statistically significant.

The forest plot shown in Figure 3 is the graphical representation of the meta-analysis for the proportion of COVID-19 patients who do not smoke. The results in Table 2 and Figure 3 show the individual study's proportion of the outcome variable and a 95% Confidence Interval. In addition, an overall pooled estimate for the study variable is also presented. The overall pooled estimate of a total sample size for 34 studies of a study variable “COVID-19 infected patients who do not smoke” is 0.88, obtained using a fixed effect model with a 95% Wald Confidence Interval. This means 88% of COVID-19 patients across the globe do not smoke. *I*^2^ statistic describes 0% of the total variation, meaning no heterogeneity exists. The last column in Table 2 and Figure 3 show the weight for each study. Moreover, the combined effect is also statistically significant (p < 0.05). The confidence intervals on the right side of zero show that the studies have a statistically significant positive effect. The confidence interval of the combined effect size does not include zero, showing that the combined effect is statistically significant.

## 4. Discussion

The main objective of this study is to find an association between smoking history and the prevalence of COVID-19 or to find if the smoking habit increases the prevalence of COVID-19. The computed results show that non-smokers have a more significant combined effect than smokers, which means smoking habit does not affect the progression of Coronavirus. Fixed effect Meta-analysis has been carried out and no heterogeneity was found. The value of *I*^2^ computed 0% for both of the analyses which means no heterogeneity was detected. Thirty-four studies were included in the analysis to check the prevalence of COVID-19 in smokers vs non-smokers. The results seem pretty interesting. The combined pooled proportions of smokers and non-smokers are 12% and 88%, respectively. The combined effects are also in this favor and found statistically significant (p < 0.05). The forest plots are graphically representing the results. The 95% Wald confidence intervals for smokers are (0.11 and 0.12), and for non-smokers they are (0.88 and 0.89) since both are entirely on the right side of zero, which depict that they contribute positive effects to the study.

The computed pooled effect of smokers in this study is 12%, close to the individual effect sizes (13%) and (14%), respectively [1],[36]. In both studies, categorical variables were summarized as counts and percentages. The computed pooled effect of non-smokers in this study is 88%, close to individual effect sizes (87%) and (90%), respectively [36],[37]. In both studies, categorical variables were summarized as counts and percentages.

## 5. Conclusions

The Meta-analysis results show that smoking habit does not play a vital role in the prevalence of COVID-19. Since the pooled proportion of smokers is 12% and the pooled proportion of non-smokers is 88%. This shows that the prevalence of COVID-19 does not increase with having a smoking habit or being a smoker. The value of *I*^2^ statistic for smokers and non-smokers proves that there is no heterogeneity in shortlisting the articles. Most of the studies were published in China, and few studies included were other than published in China, so 0% heterogeneity was detected.

Furthermore, all the individual confidence intervals are entirely on the right side of zero, and this shows that all studies have a statistically significant positive effect in this study. The confidence interval of smokers and non-smokers of the combined effect size does not include zero, which shows that the computed combined effects are statistically significant. Moreover, the combined effects for smokers and non-smokers are also statistically significant (p < 0.05). This adds value to our study that smoking does not affect the progression of COVID-19.

After obtaining the pooled estimates, it can be stated that the prevalence of COVID-19 is more in non-smokers than in smokers. The available studies incorporated into the study show that smokers are at a lower risk of facing worse health issues due to COVID-19 than non-smokers. Still, it is very important to know that smoking is associated with a variety of bad health outcomes. COVID-19 is a highly infectious virus that spreads around rapidly. Smoking is one of the significant risk factors for illness, and the ones who smoke need to continue practicing the protocols and guidelines specified by WHO [38].

Vaccination plays a vital role in competing with the severe infection in the body caused by the contagious virus. It is encouraged to get vaccinated against COVID-19 for both non-smokers and smokers. Vaccination helps resist and compete with the virus that helps the individual from hospitalization, severe illness and mortality due to COVID-19, irrespective of the individual's smoking status. It greatly emphasizes the significance of vaccination in reducing and controlling the spread of the virus. Increasing vaccination coverage is necessary to prevent further waves of viruses, infections and mortalities. Policymakers need to prioritize the importance of getting vaccinated and increasing vaccine coverage to resist and reduce the worse outcomes of the pandemic.

It is also emphasized that vaccines are distributed and available everywhere, especially in countries with low income. This could be possible with the help of effective governance and policy-making to ensure equitable and efficient distribution of vaccines. Timely access to vaccines for all populations is important.

Air pollution also plays a vital role in transmitting COVID-19 by worsening respiratory diseases. It weakens the immune system. So, it is very important to make efforts to reduce air pollution, especially in areas where the transmission of COVID-19 is at a higher rate, which will help to control the bad impact of the virus.

Some of the limitations of the study are: First, it is the comparison of the Prevalence of COVID-19 between current smokers vs non-smokers, so the patient with no smoking history is considered a non-smoker, but his history of smoking is not considered how long he has quit it; Second, the use of tobacco is also not considered in the study; Third, the other confounding factors that are influencing towards infection are also not examined in this study.
